# Management of Acute Myeloid Leukemia in a Dialysis-Dependent Patient: A Case Report, Literature Review, and Therapeutic Considerations

**DOI:** 10.7759/cureus.110774

**Published:** 2026-06-13

**Authors:** Suhail Sapkota, Alfarooq Alshaikhli, Niket Shah, Ashish Nepal, Ankit Kulkarni, Pragya Bhandari, Santosh Sapkota, Jerry Kenmoe, Maha Bayya, Shiva Shrotriya, Shailendra Sharma

**Affiliations:** 1 Internal Medicine, University of Michigan Health Sparrow, Lansing, USA; 2 Hematology and Oncology, Karmanos Cancer Institute at McLaren Greater Lansing, Lansing, USA; 3 Internal Medicine, Michigan State University, Lansing, USA; 4 Internal Medicine, WellSpan York Hospital, York, USA; 5 Internal Medicine, Cape Fear Valley Medical Center, Fayetteville, USA; 6 Internal Medicine, McLaren Healthcare Corporation, Flint, USA; 7 Nephrology, University of Michigan Health Sparrow, Lansing, USA

**Keywords:** acute myeloid leukemia, aml relapse, azacitidine, dialysis-dependent aml, end-stage renal disease, hemodialysis, hypomethylating agents, tumor lysis syndrome, venetoclax

## Abstract

Acute myeloid leukemia (AML) in patients with end-stage renal disease (ESRD) requiring hemodialysis presents numerous challenges to clinicians, including selection of appropriate chemotherapy agents, safe dose adjustment, and supportive care management. Due to the lack of prospective studies in patients undergoing dialysis, it is difficult to determine the optimal therapeutic approach for this population. These patients are commonly excluded from large-scale AML clinical trials, which further complicates the determination of effective management strategies and associated toxicities. We present the case of a 51-year-old man with ESRD on hemodialysis who developed an adverse-risk AML (characterized by a BCL6 corepressor (BCOR) mutation with runt-related transcription factor 1 (RUNX1) and nuclear receptor-binding SET domain protein 1 (NSD1) abnormalities). Initial presentation was notable for profound pancytopenia without circulating blasts, although peripheral blood flow cytometry confirmed AML. Management required a multidisciplinary discussion with leukemia specialists, nephrology, and pharmacy. The patient was treated with azacitidine and venetoclax rather than intensive induction chemotherapy because of dialysis dependence, cardiovascular comorbidities, and anticipated toxicity. Azacitidine was administered after dialysis sessions, while venetoclax dosing was adjusted because of concomitant posaconazole prophylaxis. Repeat molecular testing no longer detected previously identified BCOR and RUNX1 abnormalities, although a persistent NSD1 variant remained detectable, and molecular remission could not be confirmed. Treatment was successfully continued through 12 cycles without clinically significant tumor lysis syndrome. Approximately one year after diagnosis, relapsed AML was identified on peripheral blood flow cytometry. This case highlights the feasibility of venetoclax-based lower-intensity therapy in selected dialysis-dependent AML patients while underscoring the persistent therapeutic limitations and adverse prognosis associated with relapsed disease in this population.

## Introduction

Acute myeloid leukemia (AML) is an aggressive hematologic malignancy characterized by clonal proliferation of immature myeloid precursors leading to progressive bone marrow failure. Despite advances in molecular risk stratification and targeted therapy, outcomes remain poor in medically complex patients with significant comorbid disease [1,2]. Renal dysfunction is frequently encountered in AML; however, AML arising in patients with end-stage renal disease (ESRD) requiring chronic hemodialysis remains uncommon. The incidence of AML in dialysis-dependent patients is not well-established, largely because this population is frequently underrepresented in prospective studies [2].

Management of AML in dialysis-dependent patients presents unique clinical challenges. Intensive induction chemotherapy may result in excessive toxicity because of altered pharmacokinetics, impaired drug clearance, cardiovascular comorbidity, infectious risk, and limited physiologic reserve [3]. Patients with ESRD are largely excluded from prospective AML trials, and standardized recommendations regarding chemotherapy dosing, dialysis coordination, and supportive care remain lacking [4,5].

Lower-intensity regimens incorporating hypomethylating agents (HMAs) with venetoclax have become important therapeutic options for medically unfit AML patients, although published experience in dialysis-dependent populations remains limited to case reports and small retrospective series [5-7]. This report describes the clinical course, treatment strategy, response assessment, and relapse of a dialysis-dependent patient with adverse-risk AML treated with azacitidine and venetoclax while also reviewing available literature regarding AML management in patients requiring chronic hemodialysis.

## Case presentation

A 51-year-old man with ESRD on thrice-weekly hemodialysis for approximately three years presented with progressive fatigue, malaise, and exertional dyspnea. His medical history was significant for hypertension, coronary artery disease (CAD) status post percutaneous coronary intervention with stent placement approximately two months prior to AML diagnosis, heart failure with preserved ejection fraction (HFpEF), and chronic kidney disease (CKD) progressing to dialysis dependence. Hemodialysis was performed on a Monday/Wednesday/Friday schedule. His baseline performance status was Eastern Cooperative Oncology Group (ECOG) 1. His home medications included aspirin, carvedilol, amlodipine, hydralazine, atorvastatin, and phosphate binders. No prior exposure to cytotoxic chemotherapy, HMAs, or leukemogenic therapies was identified.

Initial laboratory evaluation demonstrated severe pancytopenia with a white blood cell count of 0.5×10³/µL, hemoglobin of 8.5 g/dL, a platelet count of 87×10³/µL, and an absolute neutrophil count (ANC) of approximately 0.1×10³/µL. Macrocytosis was present with a mean corpuscular volume (MCV) of 104.8 fL and an elevated red cell distribution width of 15.8%. Ferritin was elevated at 1600 ng/mL, while vitamin B12, folate, and copper levels were within normal limits. Peripheral blood smear demonstrated pancytopenia with macrocytic normochromic anemia, anisocytosis, severe neutropenia, thrombocytopenia, and absence of circulating blasts. No clinical or laboratory evidence of tumor lysis syndrome (TLS) was present at diagnosis.

Peripheral blood flow cytometry demonstrated approximately 32% myeloblasts characterized by dim CD45 expression and low side scatter. Blasts expressed CD13, CD33, CD34, CD38, CD117, CD123, human leukocyte antigen-DR (HLA-DR), and cytoplasmic myeloperoxidase (MPO) while lacking lymphoid lineage markers. Bone marrow biopsy performed at diagnosis demonstrated AML with approximately 28% myeloblasts. Flow cytometry showed an immature myeloid phenotype with aberrant CD7 expression. Conventional cytogenetics demonstrated a normal male karyotype (46,XY). AML fluorescence in situ hybridization (FISH) studies were negative. Myeloid molecular testing identified a BCL6 corepressor (BCOR) mutation with runt-related transcription factor 1 (RUNX1) and nuclear receptor-binding SET domain protein 1 (NSD1) abnormalities, consistent with adverse-risk AML. Serial hematologic trends during treatment are summarized in Table 1.

**Table 1:** CBC trends during treatment course AML: acute myeloid leukemia; ANC: absolute neutrophil count; PRBC: packed red blood cells; WBC: white blood cell; CBC: complete blood count; ELN: European LeukemiaNet

Timepoint	WBC (×10³/µL)	Hemoglobin (g/dL)	Platelets (×10³/µL)	ANC (×10³/µL)	Clinical course
Diagnosis (baseline)	0.5×10³/µL	8.5 g/dL	87×10³/µL	0.1×10³/µL	Severe pancytopenia at AML presentation
Post-cycle 1 (month 1)	1.2×10³/µL	7.9 g/dL	54×10³/µL	0.2×10³/µL	Persistent cytopenias with residual AML on marrow biopsy
Response assessment (month 4)	3.8×10³/µL	10.4 g/dL	142×10³/µL	1.6×10³/µL	Complete remission according to ELN criteria with hematologic recovery
Relapse (month 13)	0.7×10³/µL	5.9 g/dL	11×10³/µL	0.1×10³/µL	Recurrent pancytopenia with relapsed AML confirmed by peripheral blood flow cytometry; required PRBC transfusions

Given the patient's dialysis dependence, cardiovascular comorbidities, and anticipated toxicity associated with intensive induction chemotherapy, management was reviewed in a multidisciplinary discussion involving hematology, nephrology, oncology pharmacy, and tertiary leukemia consultants. Therapeutic strategies considered included conventional "7+3" (cytarabine + daunorubicin), lower-intensity azacitidine with venetoclax, and hybrid approaches. Concerns regarding altered pharmacokinetics, ineligibility for intensive induction therapy, infectious complications, prolonged cytopenias, and treatment-related morbidity ultimately favored the initiation of azacitidine and venetoclax.

Treatment was initiated shortly after diagnosis. Azacitidine was administered at 75 mg/m² subcutaneously on days 1-7 of a 28-day cycle and scheduled after hemodialysis sessions on dialysis days. Venetoclax was administered on days 1-21 and was not coordinated around dialysis because of minimal renal elimination. Because the patient was receiving posaconazole prophylaxis, venetoclax dosing was adjusted using a ramp-up schedule of 10 mg on day 1, 20 mg on day 2, 50 mg on day 3, and 70 mg daily thereafter.

Supportive care included antimicrobial prophylaxis with acyclovir, levofloxacin, and posaconazole. Levofloxacin and acyclovir were dose-adjusted for dialysis. TLS prophylaxis included close electrolyte monitoring, dialysis coordination, and cautious intravenous hydration because of ESRD (Table 2). Daily complete blood count, comprehensive metabolic panel, magnesium, phosphorus, and uric acid monitoring were performed during treatment initiation. No clinically significant TLS developed.

**Table 2:** Dialysis-specific chemotherapy and supportive care adjustments SQ: subcutaneous; ESRD: end-stage renal disease; CBC: complete blood count; CMP: comprehensive metabolic panel; IV: intravenous; TLS: tumor lysis syndrome; PRBC: packed red blood cells

Medication/supportive care	Regimen/dose	Dialysis considerations	Clinical notes
Azacitidine	75 mg/m² SQ days 1-7 every 28 days	Administered after hemodialysis on dialysis days	Continued through 12 cycles
Venetoclax	Days 1-21	No dialysis timing adjustment required	Minimal renal elimination
Venetoclax ramp-up	10 mg → 20 mg → 50 mg → 70 mg daily	Dose adjusted because of concomitant posaconazole	Ramp-up performed during cycle 1
Posaconazole	300 mg daily	No dialysis adjustment required	Antifungal prophylaxis
Levofloxacin	Dialysis-adjusted dosing	Administered every other day in ESRD	Antibacterial prophylaxis
Acyclovir	Reduced-dose prophylaxis	Dose adjusted for dialysis	Antiviral prophylaxis
Ondansetron	8 mg prior to azacitidine	No dialysis adjustment required	Antiemetic prophylaxis
TLS prophylaxis	Electrolyte monitoring, dialysis coordination, cautious IV hydration	Dialysis-assisted electrolyte control	No clinically significant TLS observed
Laboratory monitoring	Daily CBC, CMP, magnesium, phosphorus, uric acid during induction	Coordinated with the dialysis schedule	Close monitoring during venetoclax initiation
Transfusion support	PRBC transfusions during relapse admission	Coordinated with the hemodialysis schedule when needed	Frequent transfusion during relapse and remission period

Repeat bone marrow biopsy performed approximately one month after treatment initiation demonstrated hypocellular residual AML with marrow cellularity of 10-20% and approximately 10-12% blasts identified by CD34 and CD117 immunohistochemistry. Aspirate blast quantification was limited by hemodilution and hypocellularity but estimated approximately 15% blasts. Repeat cytogenetics remained normal, and AML FISH studies were negative.

Follow-up bone marrow evaluation approximately four months after diagnosis demonstrated findings consistent with complete remission according to the European LeukemiaNet (ELN) response criteria, including less than 5% blasts, absence of morphologic or immunophenotypic evidence of AML, recovery of neutrophil count (ANC: 1.6×10³/µL), and platelet recovery (142×10³/µL). Cytogenetics and AML FISH studies remained normal. Although BCOR and RUNX1 abnormalities were no longer detected, a persistent NSD1 variant remained detectable at a variant allele frequency of 50.5%. Because measurable residual disease (MRD) testing by validated AML-specific methodologies was not performed and the significance of the persistent NSD1 variant remained uncertain, molecular remission could not be confirmed.Bone marrow, cytogenetic, and molecular findings throughout the disease course are summarized in Table 3.

**Table 3:** Bone marrow, cytogenetic, and molecular findings throughout the disease course AML: acute myeloid leukemia; CD: cluster of differentiation; MPO: myeloperoxidase; FISH: fluorescence in situ hybridization; VAF: variant allele frequency; RUNX1: runt-related transcription factor 1; BCOR: BCL6 corepressor; NSD1: nuclear receptor-binding SET domain protein 1; HLA-DR: human leukocyte antigen-DR

Timepoint	Bone marrow findings	Flow cytometry findings	Cytogenetics/FISH	Molecular findings
Baseline (diagnosis)	AML with ~28% myeloblasts	Immature myeloid phenotype with aberrant CD7 expression	Normal male karyotype (46,XY); AML FISH negative	BCOR mutation with RUNX1 and NSD1 abnormalities
Month 1 (post-cycle 1)	Hypocellular residual AML; 10-20% cellularity with ~10-12% blasts by CD34/CD117 immunohistochemistry	Persistent AML	Cytogenetics remained normal; AML FISH negative	Persistent residual disease
Month 4 (response assessment)	Markedly hypocellular marrow (5-10%) with decreased trilineage hematopoiesis and no definitive morphologic AML	No definitive immunophenotypic evidence of AML	Normal cytogenetics; AML FISH negative	BCOR and RUNX1 abnormalities no longer detected; persistent NSD1 variant (VAF 50.5%)
Month 13 (relapse)	Bone marrow biopsy limited by hemodilution and inadequate hematopoietic sampling	Approximately 32% circulating myeloblasts expressing CD13, CD33, CD34, CD38, CD117, CD123, HLA-DR, cytoplasmic MPO	Pending	Pending

The patient continued azacitidine-based therapy through 12 cycles with outpatient dialysis coordination. Treatment was generally well-tolerated with intermittent delays related to cytopenias. The patient required frequent transfusions during the remission period. However, no episodes of febrile neutropenia or major infectious complications occurred.

Approximately 13 months after diagnosis, the patient was admitted with worsening anemia, neutropenia, and concern for gastrointestinal bleeding requiring packed red blood cell transfusion support. Computed tomography angiography (CTA) of the abdomen and pelvis demonstrated no active gastrointestinal hemorrhage but revealed colonic thickening suggestive of colitis and bladder wall thickening concerning for cystitis. Peripheral blood flow cytometry confirmed relapsed AML with approximately 32% circulating myeloblasts expressing CD13, CD33, CD34, CD38, CD117, CD123, HLA-DR, and cytoplasmic MPO. Bone marrow biopsy and repeat molecular studies at relapse were pending at the time of manuscript preparation. Intensive induction chemotherapy with standard 7+3 (cytarabine 100 mg/m² + 50% reduced dose of daunorubicin ~30 mg/m²) was considered during multidisciplinary reevaluation, although ongoing treatment planning remained complicated by ESRD, cardiovascular comorbidity, and relapsed disease status.

## Discussion

Clinical challenges of AML in ESRD

AML occurring in dialysis-dependent patients represents a major therapeutic challenge because prospective evidence guiding treatment decisions remains extremely limited. Patients with ESRD are typically excluded from AML clinical trials due to concerns regarding toxicity, altered pharmacokinetics, increased infectious risk, and competing comorbidities [1,2]. Consequently, management strategies are largely extrapolated from retrospective reports and isolated case series. Some studies that established venetoclax plus azacitidine as the standard of care for older or unfit AML patients excluded patients with severe renal impairment, leaving a critical evidence gap for this population [1,3].

Dialysis-dependent AML patients frequently have impaired physiologic reserve, cardiovascular disease, chronic inflammation, anemia, platelet dysfunction, and heightened susceptibility to infection [2]. These factors complicate both intensive induction chemotherapy and supportive care management. In the present case, multidisciplinary coordination among hematology, nephrology, and oncology pharmacy teams was essential in balancing leukemia-directed therapy against anticipated treatment-related toxicity.

Chemotherapy pharmacokinetics in dialysis

Chemotherapy administration in ESRD requires careful consideration of renal clearance and drug-related toxicities. Cytarabine-based induction regimens may present substantial toxicity concerns in dialysis-dependent patients, including prolonged cytopenias and neurotoxicity resulting from the accumulation of inactive metabolites. The National Comprehensive Cancer Network (NCCN) Guidelines recommend that doses of cytarabine ≥2 g/m² should be used with caution in patients with renal failure due to concern for neurotoxicity [4]. Anthracycline administration may additionally be complicated by underlying cardiovascular disease and reduced physiologic reserve, although the daunorubicin terminal half-life appears unaffected in hemodialysis patients [5].

Venetoclax pharmacokinetics are minimally dependent on renal elimination. Venetoclax is highly bound to human plasma proteins, with an unbound fraction of less than 0.01% [6]. Available pharmacokinetic data demonstrated that hemodialysis does not remove venetoclax, as there was no difference observed between arterial and venous samples during dialysis [7]. The Food and Drug Administration (FDA) label mentions that no dose adjustment is recommended for patients with mild, moderate, or severe renal impairment with a creatinine clearance (CLcr) of ≥15 mL/min, although the effect of ESRD (CLcr: <15 mL/min) or dialysis on venetoclax pharmacokinetics was previously listed as unknown [6]. Concomitant administration with strong CYP3A4 inhibitors such as posaconazole requires substantial dose reduction, as strong CYP3A inhibitors decrease venetoclax clearance by approximately 84% [8,9]. In this patient, venetoclax ramp-up dosing was modified appropriately during azole prophylaxis.

Azacitidine pharmacokinetics in dialysis-dependent patients remain less well-defined. It undergoes spontaneous hydrolysis and deamination mediated by cytidine deaminase rather than renal elimination, with less than 2% of the dose recovered unchanged in urine [10]. Existing guidance suggests azacitidine may be administered without major dose adjustment, although administration after dialysis sessions is generally recommended to minimize premature clearance [10]. In our case, azacitidine was consistently administered after hemodialysis and remained clinically feasible over prolonged treatment.

Literature review of reported dialysis-dependent AML cases

To identify previously reported cases of AML in dialysis-dependent patients, a focused narrative literature review was performed using PubMed/MEDLINE and Google Scholar through May 2026. Search terms included "acute myeloid leukemia", "AML", "hemodialysis", "dialysis", "end-stage renal disease", "ESRD", "venetoclax", "azacitidine", and "renal impairment". English-language case reports, retrospective studies, pharmacokinetic investigations, and case series describing AML treatment in dialysis-dependent patients were reviewed. Reports lacking sufficient clinical treatment information or involving hematologic malignancies other than AML were excluded. Given the limited number of published reports, a narrative rather than a systematic review approach was used.

Among reports of intensive chemotherapy, Krashin et al. reported a 54-year-old dialysis-dependent AML patient treated with modified induction using dose-adjusted daunorubicin and cytarabine. Pharmacokinetic analysis suggested that while hemodialysis did not significantly alter daunorubicin terminal half-life, systemic drug exposure remained substantial, supporting cautious dose modification and close toxicity monitoring [5]. Perry et al. reported successful allogeneic bone marrow transplantation in a patient with AML and pre-existing ESRD using high-dose cyclophosphamide with hemodialysis support, demonstrating that pharmacokinetic-guided conditioning is feasible in this population [11]. Kourelis et al. reported a dialysis-dependent AML patient treated with daunorubicin and cytarabine induction followed by decitabine maintenance, although relapse occurred within five months [12].

Naka et al. described the feasibility of venetoclax and azacitidine therapy in 15 AML patients with severe renal impairment (eGFR: <30 mL/min), including patients on hemodialysis, demonstrating that most patients achieved favorable responses and renal function generally remained stable or improved during treatment. This study also emphasized the importance of TLS monitoring [13]. Akagi et al. described the successful treatment of dialysis-dependent AML using azacitidine and venetoclax, although the patient later died from septic complications despite a hematologic response [14]. Krüger et al. conducted a multicenter retrospective study of 130 newly diagnosed older AML patients receiving HMA/venetoclax and found that while pre-existing CKD showed a trend toward lower overall response rates (OR: 0.5; p=0.07), it did not significantly affect overall survival; however, acute kidney injury during treatment was an independent prognostic factor for inferior overall survival (HR: 1.86; p=0.01) [15].

Compared with previously reported cases, our patient demonstrated prolonged outpatient disease control extending approximately 13 months despite adverse-risk molecular features and dialysis dependence. The median overall survival with venetoclax plus azacitidine in the VIALE-A trial was 14.7 months in the overall population at a median follow-up of 20.5 months, with a composite complete remission rate of 66.4% [1]. At the long-term follow-up (median: 43.2 months), the hazard ratio for death remained significant at 0.58 (95% CI: 0.47-0.72; p<0.001), with an estimated 24-month overall survival rate of 37.5% [3]. Achieving 13 months of disease control in a dialysis-dependent patient with adverse-risk features is therefore notable, though it falls within the expected range for the general venetoclax-azacitidine population. This case additionally provides longitudinal molecular data, detailed dialysis coordination, and prolonged follow-up through relapse. Published reports and our case describing AML management in dialysis-dependent patients are summarized in Table 4.

**Table 4:** Published reports and present case summary of AML management in dialysis-dependent patients AML: acute myeloid leukemia; ESRD: end-stage renal disease; HD: hemodialysis; PK: pharmacokinetics; CR: complete remission; CR2: second complete remission; BMT: bone marrow transplantation; TBI: total body irradiation; TLS: tumor lysis syndrome; HAM: high-dose cytarabine and mitoxantrone; HMA: hypomethylating agent; RI: renal impairment; eGFR: estimated glomerular filtration rate; ORR: overall response rate; AKI: acute kidney injury; OS: overall survival; FIA: fludarabine, idarubicin, and cytarabine; VAF: variant allele frequency; PO: oral administration; CIVI: continuous intravenous infusion; t½: terminal half-life; AUC: area under the curve; FLT3-TKD: FMS-like tyrosine kinase 3 tyrosine kinase domain mutation; RUNX1: runt-related transcription factor 1; BCOR: BCL6 corepressor; NSD1: nuclear receptor-binding SET domain protein 1; ELN:

Year	Citation	Age/sex	Renal status	Cytogenetics/molecular	Induction regimen	Consolidation/maintenance	Outcomes
1999	Perry et al. [11]	42/M	ESRD on HD	AML in CR2	High-dose cyclophosphamide (60 mg/kg/day × 2) + TBI; HD timed after CY based on PK data	Allogeneic BMT from a matched related donor	Engraftment day +22; 100% donor karyotype; remission at 3 years post-transplant
2011	Kourelis et al. [12]	66/M	ESRD on HD (post-renal transplant graft rejection)	47,XY,+10,t(11;17)(q23;q25); MLL-AF4 fusion	Daunorubicin 12 mg/m²/day IV × 3 days + cytarabine 200 mg/m²/day × 5 days CIVI; daily HD for TLS prevention	Decitabine maintenance	Relapsed after 5 months; died
2013	Labno-Kirszniok et al. [16]	38/M	ESRD on HD; von Hippel-Lindau	inv(16)(p13;q22); CBFB-MYH11	Daunorubicin 60 mg/m² days 1-3 + cytarabine 200 mg/m² days 1-5	HAM: mitoxantrone 10 mg/m² days 1-2 + cytarabine 1500 mg/m² days 1-5	Prolonged marrow aplasia → interstitial pneumonia, septicemia → died from respiratory failure
2016	Krashin et al. [5]	54/F	ESRD on chronic HD	AML (subtype not specified)	Modified induction: reduced-dose daunorubicin + cytarabine; daunorubicin t½ unaffected by HD; AUC₀₋₂₃ₕ comparable to full-dose patients	Not described	PK analysis supports cautious dose modification in HD patients
2017	Benitez et al. [17]	19/M	ESRD on intermittent HD	AML (relapsed/refractory)	Clofarabine-based regimen with dose adjustment for HD	Not described	HD did not reliably remove clofarabine; dose adjustment required
2019	Tollkuci et al. [18]	51/M and 65/F	ESRD on HD; FLT3-positive	FLT3-mutated AML	Standard induction + midostaurin	Not described	Midostaurin PK described; tolerated despite HD
2021	Tollkuci et al. [19]	68/F	ESRD on HD; FLT3-TKD+	FLT3-TKD mutation	7+3 (cytarabine + idarubicin); day 14 BMBx leukemia-free	Gilteritinib 120 mg PO daily (relapsed AML)	Managed on HD; infections complicated course
2022	Edahiro et al. [20]	49/M	ESRD on HD	Normal karyotype 46,XY; NPM1−, FLT3-ITD−, CEBPA−	Standard 7+3 (cytarabine + daunorubicin) → CR; 4 courses consolidation	Azacitidine + venetoclax (salvage after relapse at 9 months)	Deemed ineligible for transplant
2023	Akagi et al. [14]	73/M	ESRD on HD	inv(16)(p13.1;q22); CBFB-MYH11	Azacitidine + venetoclax	Continued azacitidine + venetoclax	Hematologic response; died from *E. coli *sepsis at 12 months
2023	Naka et al. [13]	Cohort (n=15; subset HD)	Severe RI (eGFR 30), including HD patients	Variable	Venetoclax + azacitidine	Not described	Most achieved favorable responses; renal function stable/improved; TLS risk emphasized
2025	Herstein et al. [21]	65/F	Chronic ESRD on intermittent HD	Newly diagnosed AML	FIA + venetoclax	Not described	Achieved complete remission; demonstrates feasibility of intensive + venetoclax in HD
2025	Krüger et al. [15]	Multicenter cohort (n=130; subset CKD)	Pre-existing CKD (subset with severe RI)	Variable	HMA + venetoclax	Variable	Pre-existing CKD: trend toward lower ORR (OR: 0.5; p=0.07); AKI during therapy: independent predictor of inferior OS (HR: 1.86; p=0.01)
2026	Present case	51/M	ESRD on HD since 2022 (M/W/F)	Normal karyotype; BCOR, RUNX1, NSD1 abnormalities (adverse-risk)	Azacitidine 75 mg/m² SQ days 1-7 + venetoclax days 1-21 (ramp-up 10→20→50→70 mg with posaconazole)	Continued azacitidine + venetoclax × 12 cycles	Achieved CR per ELN criteria with disappearance of BCOR and RUNX1 abnormalities; persistent NSD1 variant (VAF 50.5%); relapse identified approximately 13 months after diagnosis

Therapeutic selections in dialysis-dependent AML

Therapeutic selection in dialysis-dependent AML must account for disease biology, performance status, comorbidity burden, transplant eligibility, and anticipated toxicity. The NCCN Guidelines recommend azacitidine plus venetoclax (category 1) or decitabine plus venetoclax as preferred lower-intensity regimens for patients who are not candidates for intensive induction therapy [4]. Intensive induction chemotherapy may provide higher remission rates in selected patients but is frequently associated with substantial morbidity in ESRD [2].

Responses to venetoclax-based therapy may deepen gradually over multiple cycles, and residual disease after cycle 1 should not necessarily be interpreted as treatment failure in clinically stable patients. The NCCN Guidelines note that response may not be evident before 3-4 cycles of treatment with HMAs and venetoclax combination regimens may be continued for patients whose disease demonstrates clinical improvement [4]. Residual AML persisted following cycle 1 but subsequently evolved into complete remission according to ELN criteria, accompanied by the normalization of cytogenetic studies and clearance of previously detected BCOR and RUNX1 abnormalities, although molecular remission could not be confirmed because MRD testing was unavailable and the NSD1 variant persisted [22].

Supportive care alone may be appropriate in highly frail patients or those unable to tolerate leukemia-directed therapy. However, Venditti et al. suggest that patients benefit from venetoclax plus azacitidine regardless of age or degree of frailty as assessed by the AML composite model, supporting the consideration of active therapy even in medically complex patients [23]. Pharmacologic considerations are summarized in Table 5.

**Table 5:** Summary of pharmacological recommendations for AML therapy in ESRD AML: acute myeloid leukemia; ESRD: end-stage renal disease; PK: pharmacokinetics; HD: hemodialysis; HMA: hypomethylating agent; CLcr: creatinine clearance; CYP3A4: cytochrome P450 3A4; CL/F: apparent clearance; AKI: acute kidney injury; OS: overall survival; BUN: blood urea nitrogen; AEs: adverse events; RI: renal impairment; FDA: Food and Drug Administration; AUC: area under the curve; Cmax: maximum concentration; fu: unbound fraction; t½: terminal half-life

Drug class	Agent	Renal elimination	PK in ESRD/HD	Dose adjustment in ESRD	Key considerations
BCL-2 inhibitor	Venetoclax [6]	0.1% urinary excretion; >99.9% fecal	ESRD/HD do not alter unbound Cmax or AUC; fu ~2-fold higher in ESRD but unbound exposure comparable; HD does not remove drug	No adjustment required (CLcr ≥15 mL/min); dedicated PK study supports use in ESRD/HD	Strong CYP3A4 inhibitors decrease CL/F by ~84%, requiring dose reduction; close TLS monitoring; AKI during therapy independently associated with inferior OS
HMA	Azacitidine [10]	2% urinary excretion unchanged; undergoes spontaneous hydrolysis and cytidine deaminase-mediated deamination	Severe RI (CLcr 30) increases exposure ~70% (single dose), ~41% (multiple doses); not correlated with increased AEs	No initial dose adjustment; reduce 50% if unexplained BUN/creatinine elevations; administer after HD sessions	Widely used in ESRD; administer after dialysis to optimize exposure
HMA	Decitabine [12]	Primarily eliminated by deamination via cytidine deaminase; no formal PK data in RI per FDA label	Limited ESRD data	No adjustment usually required	Monitor for myelosuppression; case series support safe use
FLT3 inhibitor (type I)	Midostaurin [18]	5% urinary; 95% fecal (4% unchanged); metabolized by CYP3A4; >99.8% protein bound	No clinically meaningful PK effects with CLcr ≥30; PK in severe RI (CLcr 15-29) unknown	No formal adjustment for CLcr ≥30; PK in ESRD unknown	Case reports describe use in HD patients; t½ 19 hours (parent), 482 hours (CGP52421 metabolite)
FLT3 inhibitor (type I)	Gilteritinib [19]	16.4% urinary (as unchanged + metabolites); 64.5% fecal; metabolized by CYP3A4; 94% protein bound	No clinically meaningful PK effects with mild-moderate RI (CLcr 30-80); effect of severe RI (CLcr ≤29) unknown	No formal adjustment; monitor QTc	Strong CYP3A4 inhibitors increase AUC by ~120%; t½ 113 hours; case reports describe use in HD patients
Anthracycline	Daunorubicin [5]	Limited renal excretion; primarily hepatobiliary	Terminal t½ unaffected by HD; estimated AUC comparable to full-dose patients at reduced doses	Dose reduction recommended (case-based evidence)	Monitor cardiac function; increased toxicity risk in ESRD
Anthracycline	Idarubicin [19]	Similar to daunorubicin; increased exposure expected in RI	Limited data	25% dose reduction in severe RI	Monitor closely; used in reduced doses in case reports

TLS and dialysis coordination

TLS remains a major concern during venetoclax initiation, particularly in patients with renal dysfunction. The FDA label specifies that patients with reduced renal function (CLcr: <80 mL/min) require more intensive prophylaxis and monitoring to reduce TLS risk [6]. The NCCN Guidelines recommend inpatient treatment during the first cycle, especially through dose escalation, with blood chemistry monitoring every 6-8 hours after initiation for 24 hours following maximum dose escalation [4]. In AML, the venetoclax ramp-up schedule is 100 mg on day 1, 200 mg on day 2, and 400 mg from day 3 onward [1].

Notably, this patient did not develop clinically significant TLS despite dialysis dependence. Multiple factors likely contributed, including low baseline leukemic burden, careful venetoclax dose ramp-up, intensive laboratory monitoring, and close dialysis coordination. Hemodialysis scheduling additionally requires coordination with chemotherapy administration. Venetoclax administration was not timed around dialysis because of limited dialyzability, whereas azacitidine was administered following dialysis sessions to optimize drug exposure [7].

Infectious and bleeding complications

Dialysis-dependent AML patients remain highly susceptible to infections and bleeding complications because of neutropenia, platelet dysfunction, vascular access requirements, and comorbid disease burden. The NCCN Guidelines recommend posaconazole for antifungal prophylaxis, noting it significantly decreases fungal infections compared to fluconazole and itraconazole [4]. This patient received prophylactic acyclovir, levofloxacin, and posaconazole throughout therapy. Although recurrent cytopenias occurred, major infectious complications and febrile neutropenia were not observed during the remission period.

At relapse, the patient developed worsening anemia and concern for gastrointestinal bleeding requiring blood transfusion. Imaging demonstrated colitis without active hemorrhage, emphasizing the complexity of supportive care management in relapsed AML with ESRD.

Prognostic considerations and molecular biology

BCOR and RUNX1 mutations are classified as one of the adverse-risk markers in the ELN 2022 risk stratification system [4,24]. BCOR mutations are associated with inferior overall survival and lower complete remission rates with intensive chemotherapy [25]. RUNX1 mutations, found in approximately 10% of AML patients, are associated with older age, more immature morphology, and inferior event-free, relapse-free, and overall survival [26]. Notably, BCOR and RUNX1 mutations frequently co-occur, and the combination has been associated with increased risk of post-transplant relapse [25].

The interpretation of the persistent NSD1 variant requires caution. Although BCOR and RUNX1 abnormalities were no longer detected during remission, the NSD1 variant persisted at a variant allele frequency of 50.5%. Persistence of a molecular alteration at approximately 50% variant allele frequency may reflect several biologic possibilities, including residual leukemic clonality, clonal hematopoiesis, or a germline variant. Because matched germline testing was not performed and relapse molecular profiling remained unavailable at the time of manuscript preparation, the precise significance of the persistent NSD1 variant could not be determined. Therefore, the finding should not be interpreted as definitive evidence of residual AML or molecular relapse.

Similarly, disappearance of BCOR and RUNX1 abnormalities should not be equated with a confirmed molecular remission because validated MRD testing was not performed. Rather, these findings suggest molecular clearance of previously detected abnormalities while leaving uncertainty regarding residual disease burden below the limit of detection.

The subsequent relapse approximately 13 months after diagnosis underscores the aggressive biology of adverse-risk AML and the therapeutic challenges faced by medically complex patients who are not candidates for allogeneic stem cell transplantation.

Clinical lessons and future directions

Allogeneic stem cell transplantation remains the only curative option for adverse-risk AML, yet dialysis-dependent patients face formidable barriers to transplant eligibility, including impaired physiologic reserve, conditioning regimen toxicity, and the logistical complexity of peri-transplant dialysis management [27].

Several important practical considerations emerged from this case regarding the management of dialysis-dependent AML. First, venetoclax-based lower-intensity therapy may remain feasible in selected ESRD patients with careful dialysis coordination and antimicrobial prophylaxis [7]. Second, azacitidine can be administered without initial dose adjustment in renal impairment, though administration after dialysis sessions is recommended to optimize exposure [10]. Third, residual disease after early treatment cycles should be interpreted cautiously because delayed responses may occur with HMA and venetoclax therapy [4]. Fourth, TLS risk can be effectively mitigated in dialysis-dependent patients through careful venetoclax dose ramp-up, intensive monitoring, and dialysis coordination [4]. Finally, multidisciplinary collaboration among hematology, nephrology, pharmacy, and supportive care teams remains essential in optimizing outcomes.

An important limitation of this report is that bone marrow, cytogenetic, and molecular studies obtained at relapse remained pending at the time of manuscript preparation. Although relapse was strongly supported by peripheral blood flow cytometry demonstrating approximately 32% circulating myeloblasts, definitive characterization of clonal evolution, mutation acquisition, mutation persistence, or cytogenetic progression could not be performed. Consequently, any association between the persistent NSD1 variant and subsequent relapse remains speculative and should be interpreted cautiously.

## Conclusions

AML management in dialysis-dependent patients requires individualized multidisciplinary decision-making because prospective evidence guiding chemotherapy selection and supportive care remains limited. Careful consideration of treatment-related toxicity, dialysis coordination, infectious risk, and comorbid disease burden is essential.

This case demonstrates the feasibility of administering azacitidine and venetoclax in a carefully selected dialysis-dependent patient with close multidisciplinary monitoring, dialysis coordination, and supportive care. The patient achieved complete remission according to ELN criteria and experienced approximately 13 months of disease control despite adverse-risk molecular features. However, frequent transfusion requirements, treatment delays, persistent molecular uncertainty, and eventual relapse highlight the ongoing therapeutic challenges in this population. Because this report reflects a single-patient experience, conclusions regarding efficacy and safety should be interpreted cautiously. Additional studies evaluating lower-intensity AML regimens in ESRD are needed.
